# Anaphylactic shock following the bite of a wild Kayan slow loris (*Nycticebus kayan*): implications for slow loris conservation

**DOI:** 10.1186/1678-9199-20-43

**Published:** 2014-10-02

**Authors:** George Madani, K Anne-Isola Nekaris

**Affiliations:** Little Fireface Project, Cisurupan, Cipaganti, Garut, Java Indonesia; Nocturnal Primate Research Group, Oxford Brookes University, Headington Campus, Gipsy Lane, Oxford, OX3 0BP UK

**Keywords:** Anaphylaxis, Hypersensitivity, Systemic reaction, Malaysia, Adrenaline, Necrosis, Paresthesia, Animal bite, Mammal venom, Hematuria

## Abstract

**Background:**

Asian slow lorises (*Nycticebus* spp.) are one of few known venomous mammals, yet until now only one published case report has documented the impact of their venomous bite on humans. We describe the reaction of a patient to the bite of a subadult *Nycticebus kayan*, which occurred in the Mulu District of Sarawak in 2012.

**Findings:**

Within minutes of the bite, the patient experienced paraesthesia in the right side of the jaw, ear and right foot. By 40 minutes, swelling of the face was pronounced. The patient was admitted to Mulu National Park Health Clinic/Klinik Kesihatan Taman Mulu Tarikh, at which time he was experiencing: swollen mouth, chest pain, mild abdominal pain, nausea, numbness of the lips and mouth, shortness of breath, weakness, agitation and the sensation of pressure in the ears due to swelling. The blood pressure was 110/76, the heart ratio was 116 and oxygen saturation was 96%. The patient was treated intramuscularly with adrenaline (0.5 mL), followed by intravenous injection of hydrocortisone (400 mg) and then intravenous fluid therapy of normal saline (500 mg). By 8 h10 the next day, the patient’s condition had significantly improved with no nausea, and with blood pressure and pulse rate stable.

**Conclusions:**

A handful of anecdotes further support the real danger that slow loris bites pose to humans. As the illegal pet trade is a major factor in the decline of these threatened species, we hope that by reporting on the danger of handling these animals it may help to reduce their desirability as a pet.

## Findings

### Introduction

Anaphylactic reactions can be particularly dangerous in remote tropical locations where medical aid is not available. Anaphylactic shock following animal bites and stings can onset within 10–15 minutes, a perilous situation when medical assistance may be absent or far away [1]. Symptoms of anaphylactic shock include welts or rash, shortness of breath, tightening of the chest, numbing or tingling of the extremities, and potential death [2]. Demain [2] describes that anaphylaxis caused by bites and stings usually occurs after previous injections of the inducing substance, followed by a suitable time period for incubation. Klotz *et al*. [3] note that an increasing number of unusual taxa are now known to induce anaphylactic shock.

One such animal is the slow loris (Primates: Lorisidae: *Nycticebus* spp.), one of few known venomous mammals [4]. These nocturnal primates are found throughout South and Southeast Asia. Their venom delivery system is unusual in that saliva must be combined with oil from a brachial gland, located in the upper arm near the elbow [5]. Threatened animals wrap their arms tightly above the head to combine the fluids, and the bite is inflicted with front teeth that deliver the venom via capillary action, a defensive pose that suggests the venom may be useful against potential predators [6]. Alterman [6] provided evidence that slow loris venom repelled predators and that the venom can be fatal to mice. Nekaris *et al*. [7] further showed that the venom seems to be used as a weapon against conspecifics, which show necrotic wounds when bitten, and that the venom can kill a variety of small-bodied animals. So far, the precise chemical structure of the venom remains unknown. The brachial gland exudate is known to contain a complex mixture of 60–212 volatile and semi-volatile compounds, including peptides similar to Fel-D1 [5].

A single medical case has been published of an adult man entering anaphylactic shock upon being bitten by the largest slow loris species (1000–2000 g), the Bengal slow loris *N. bengalensis* [8]. Otherwise healthy, the bite recipient was given relief through injections of 1 cc of 1:1,000 solution of epinephrine and 50 mg of diphenhydramine, followed by three subsequent injections of 75 mg of meperidine. The patient had been in possession of the wild-caught slow loris for 2.5 years and reported potential sensitization to the bite, having been nipped frequently [9]. Of note was that the victim introduced the animal to another wild-caught loris with which it fought, and he was bitten by his loris during separating them from the fight.

**Figure 1:**
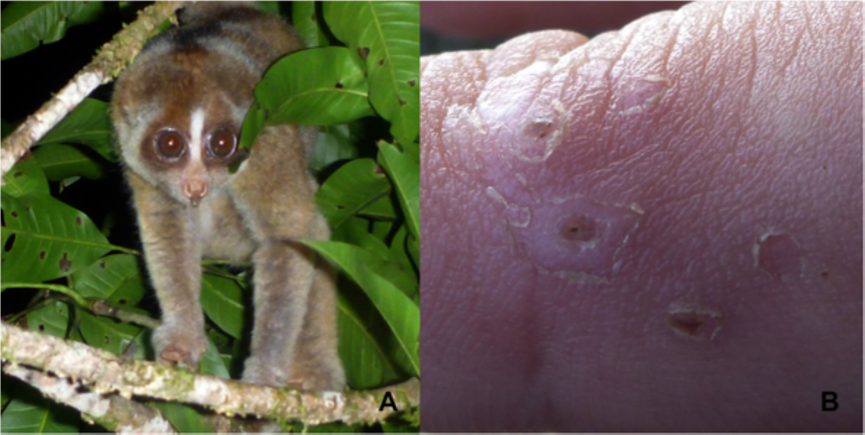
**This subadult slow loris bit the victim’s finger intensely resulting in a severe wound. (Panel A)** Subadult *Nycticebus kayan* before the victim handled it – already a large drop of saliva can be seen protruding from the animal’s mouth. **(Panel B)** The bite site 12 days after the bite. (Photos by G. Madani).

Here we report the second medically-evaluated case of anaphylactic shock by a slow loris, but with two additional aspects. First, the slow loris in question was the Kayan slow loris (*N. kayan*) from Sarawak, Malaysian Borneo. Not only is this species significantly smaller than *N. bengalensis*, weighing only 400–500 g, but the loris administering the bite was also a subadult (Figure 1). Secondly, the victim had never before touched or even seen a slow loris, meaning any reaction he would have to one’s bite would be completely naïve rather than a sensitized reaction, as has been argued to the case described by Wilde [8].

### Case report

On the 8th of April, 2012 whilst travelling and spotlighting in the Mulu district of Sarawak, Malaysian Borneo, one of the authors of the present study (GM) came across a slow loris, *N. kayan*, 2 m high in a mango tree (*Mangifera* spp.). In a combination of curiosity and foolhardiness, GM climbed the tree and in the process, the loris fell to the ground. On attempting to pick it up, the subadult individual raised its arms up over its head whilst baring its teeth in a classic defensive posture [6].

**Figure 2:**
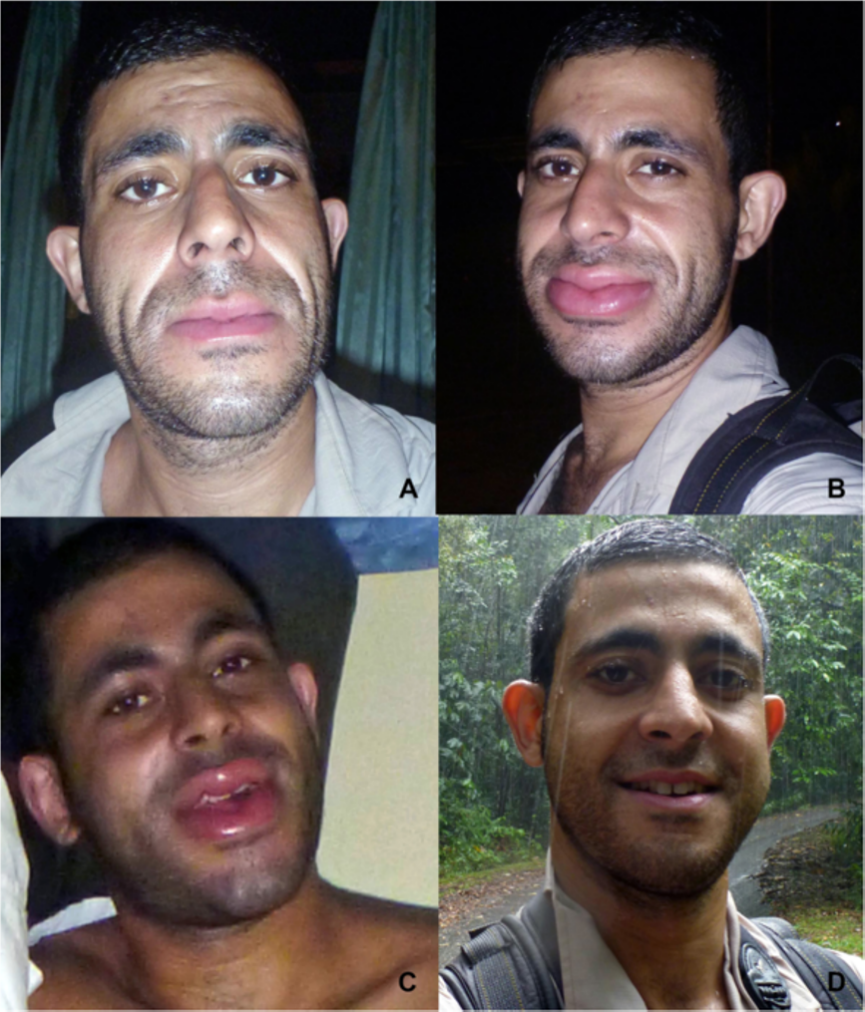
**After the bite, the patient showed extreme swelling, worsening over time. (Panel A)** 33 minutes after the bite; **(Panel B)** 54 minutes after the bite; **(Panel C)** one hour and 39 minutes after the bite; and **(Panel D)** one week after the bite (photos by G. Madani).

The head of the loris was restrained in a ‘bird grip’ (the first two fingers of the hand positioned over either side of the head whilst the rest of the hand sits over the animals back and shoulders) to prevent it from turning to bite the handler. This method proved futile and the loris subsequently turned its head and bit deeply into the middle phalanx of the middle finger on the right hand. The bite lasted for almost 30 seconds with the animal having to be forcibly extricated from the finger, whereupon it was immediately released.The bite occurred at 22 h03. Within two minutes of the bite, a sensation of paresthesia was felt in the right side of the jaw, ear and right foot. Within 33 minutes, swelling around the face had become noticeable (Figure 2A) and within 54 minutes it was pronounced (Figure 2B). The real concern at this stage was the risk respiratory obstruction and in the interim to heading to the Mulu National Park Health Clinic the patient took 20 mg of cetirizine 20 minutes after the bite.

The patient was admitted to Mulu National Park Health Clinic/Klinik Kesihatan Taman Mulu Tarikh, at 23 h00 (Figure 2C). Symptoms that were reported at the time included: swollen mouth, chest pain, mild abdominal pain, nausea, numbness of the lips and mouth, shortness of breath, weakness, agitation and the sensation of barotrauma due to swelling. The blood pressure (BP) was 110/76, the heart rate (HR) was 116 and oxygen saturation (SpO_2_) was 96%.

The patient received an intramuscular injection of adrenaline (0.5 ml) at 23 h30. At 23 h50, the doctor administered hydrocortisone (400 mg) intravenously followed by intravenous fluid therapy of normal saline (500 mg). The ECG reading showed no apparent distress (NAD). At 2 h30 the doctor took the following vitals: SPO_2_ 96%; HR 82; BP 110/80; urine output was normal. At 8 h00 the next day the BP was 130/80, SpO_2_ was 92% and HR was 83.

By 8 h10 the next day, the patient’s condition was significantly improved with no nausea, BP and pulse rate (PR) were stable, SpO_2_ 95%, no abdominal pain, middle finger still numb, no chest pain, and lip swelling reduced. The patient was prescribed: prednisolone tablets (50 mg), loratadine tablets (10 mg) and amoxicillin tablets (500 mg). Within one week, the physical characteristics of the patient had returned largely to normal (Figure 2D). The site of the bite healed well over the following weeks and showed no sign of necrosis; still after 12 days, bite marks were still noticeable (Figure 1).

### Discussion

The purpose of loris venom has been discussed in detail with several functions proposed including: defense from predators, prey neutralization, antagonistic conspecific interactions, protection from ectoparasites and Mullerian mimicry [7]. Local people have long reported that the slow loris bite is deadly and dangerous [10].

This case, the second medically evaluated case of anaphylaxis following a slow loris bite and the first reported from *N. kayan* demonstrates the effects of envenomation from these primates. In this instance, the function of the venom can be clearly demonstrated as having a negative effect and potentially fatal consequence on a person. Other unpublished accounts and stories from indigenous local people in Southeast Asia of loris bites also attest to the potency of the venom with one reported instance of death [8, 10]. Several cases of loris bites from North American and Asian zoos have required hospitalization with one patient passing blood in their urine for several weeks post bite [U Streicher, N Gibson, and J Recuero, unpublished data]. Other reported symptoms include fainting and temporary blindness [Starr, personal communication]. Recuero [11] reported being bitten by *N. pygmaeus*. He described it as quite painful, but not serious, despite several unusual symptoms: the bite became inflamed, and during three hours after the bite, the blood did not coagulate easily; for two days after the bite, blood appeared in the victim’s urine. Gibson [unpublished data] was handed over an adult *N. pygmaeus*, which had been kept as a pet for seven years. The owner reported that although she had been nipped before, on this occasion, the loris bit deeply to the bone and injected venom. She reported symptoms arising within 30 minutes including: dull sensation from the feet, front leg, and belly; facial numbness; extreme swelling of the hands and feet, and associated red rash on the legs; inability to walk and difficulty breathing. The patient reported being admitted to Samitvej Sukhumvit Hospital Bangkok at 0500, injected with adrenaline, and released at 1500 the same day. She reported swelling and pain for an additional four days.

The difference between our case, the Wilde [8] report and the anecdotes above is that in this instance the patient was immunologically naïve having never before even seen a loris, nor was he ever bitten by an animal with similar venom to a loris [5]. The patient still experienced a full systemic reaction resulting in anaphylactic shock [12]. His reaction was similar to naïve patients experiencing a systemic reaction from insect stings [13].

Unlike the captive (albeit wild-caught) loris in the Wilde [8] case, the slow loris in this instance was fully wild, thus conceivably with ready access to its natural food sources from which it could sequester the necessary compounds used to bolster the venom. As reported in the Wilde case study [8], it was shown that once the loris bit its handler, it held on for several seconds. Lorises have procumbent anterior incisors that can act as an efficient venom delivery system through their ability to draw liquid (in this case saliva mixed with brachial exudate) upwards [6]. By holding the bite for as long as possible it could potentially enable the loris to transfer as much of the venom into the antagonist as possible. Interestingly, illegal traders in Indonesia keep a bucket of water ready when handling lorises; should the loris begin to bite, the handler immediately douses it with water to force it to release its grip [6].

Of interest are several descriptions of severe necrosis following loris bites. Streicher [14] and Nekaris *et al*. [7] refer to the high instance of scars and wounds in wild animals due to possible antagonistic encounters between conspecifics. In one instance, a villager from Sumedang, West Java, reported the loss of a finger subsequent to necrosis following a bite from a Javan slow loris (*N. javanicus*), and another villager from West Java Province reported the loss of an entire arm [10]. One of us (KAIN) experienced necrosis at the bite site after being bitten through a glove by a *N. bengalensis* in Singapore; the bite took more than 20 days to heal. Whether this is due to the nature of the venom, species-specific characteristics of the venom, or consequence of poor healing conditions in the moist tropics is yet to be elucidated. We do know that slow lorises bitten in the wild suffer severe wounds that heal whereas loris wounds in captive lorises are one of the most common causes of death [15]. In the case of this report, no necrosis of the flesh occurred.

Internationally lorises are threatened with extinction due to habitat loss, hunting for traditional medicine and poaching for the illegal pet trade. Illegal pet traders, aware of the potency of loris venom, cruelly cut out the front teeth to make them more appealing pets [16]. Recent Web 2.0 videos and other media portraying loris as cute pets have further exacerbated and encouraged the serious problem of their illegal trade. By reporting on the very real risks associated with loris envenomation it is hoped that it will help curb the illegal trade in these enigmatic and threatened species.

### Consent

Written informed consent was obtained for the publication of this report and any accompanying images.

## Authors’ information

GM is a freelance wildlife ecologist based in Australia. He specializes in fauna surveys and wildlife monitoring programs across Australia and has also undertaken survey work in Southeast Asia, Central America and the Middle East. After his experience with the slow loris, he now works to raise awareness of the plight of this enigmatic species. KAIN is a Professor in Primate Conservation at Oxford Brookes University and Director of the Little Fireface Project, and international research consortium to understand and conserve Asia’s lorises.

## References

[1] Yeargin SW, Yeargin BE, Anderson JM, Casa DJ (2011). Anaphylactic shock, hypothermia, diabetes, and wilderness medicine. Preventing sudden death in sport and physical activity.

[2] Demain JG (2003). Papular urticaria and things that bite in the night. Curr Allergy Asthma Rep.

[3] Klotz JH, Klotz SA, Pinnas JL (2009). Animal bites and stings with anaphylactic potential. J Emerg Med.

[4] Ligabue-Braun R, Verli H, Carlini CR (2012). Venomous mammals: a review. Toxicon.

[5] Hagey LR, Fry BG, Fitch-Snyder H, Gursky SL, Nekaris KAI (2007). Talking defensively, a dual use for the brachial gland exudate of slow and pygmy lorises. Primate anti-predator strategies.

[6] Alterman L, Alterman L, Doyle GA, Izard MK (1995). Toxins and toothcombs: potential allospecific chemical defenses in *Nycticebus* and *Perodicticus*. Creatures of the Dark.

[7] Nekaris KAI, Moore RS, Rode EJ, Fry BG (2013). Mad, bad and dangerous to know: the biochemistry, ecology and evolution of slow loris venom. J Venom Anim Toxins incl Trop Dis.

[8] Wilde H (1972). Anaphylactic shock following bite by a slow loris, *Nycticebus coucang*. Am J Trop Med Hyg.

[9] Golden DB (2005). Insect sting allergy and venom immunotherapy: a model and a mystery. J Allergy Clin Immunol.

[10] Nijman V, Nekaris KAI (2014). Traditions, taboos and trade in slow lorises in Sundanese communities in southern Java, Indonesia. Endang Species Res.

[11] Recuero J: **Comment to - Are slow lorises really venomous?** [http://primatology.net/2010/10/19/are-slow-lorises-really-venomous/]

[12] Lockey RF (1974). Systemic reactions to stinging ants. J Allergy Clin Immunol.

[13] Biló BM, Rueff F, Mosbech H, Bonifazi F, Oude‒Elberink JN (2005). EAACI Interest group on insect venom hypersensitivity: **Diagnosis of Hymenoptera venom allergy**. Allergy.

[14] Streicher U: **Aspects of ecology and conservation of the pygmy loris*****Nycticebus pygmaeus*****in Vietnam.** *PhD Thesis* Ludwig-Maximilians Universität, München, Department of Veterinary Science; 2004

[15] Fuller G, Lukas KE, Kuhar C, Dennis PM (2014). A retrospective review of mortality in lorises and pottos in North American zoos, 1980-2010. Endang Species Res.

[16] Nekaris KAI, Campbell N, Coggins TG, Rode EJ, Nijman V (2013). Tickled to death: analysing public perceptions of ‘cute’ videos of threatened species (slow lorises – *Nycticebus* spp.) on web 2.0 Sites. PLoS One.

